# Molecular determinants of staphylococcal biofilm dispersal and structuring

**DOI:** 10.3389/fcimb.2014.00167

**Published:** 2014-11-26

**Authors:** Katherine Y. Le, Sana Dastgheyb, Trung V. Ho, Michael Otto

**Affiliations:** ^1^Pathogen Molecular Genetics Section, Laboratory of Human Bacterial Pathogenesis, National Institute of Allergy and Infectious Diseases, National Institutes of HealthBethesda, MD, USA; ^2^Division of Hospital Internal Medicine, Department of Medicine, Mayo Clinic College of MedicineRochester, MN, USA; ^3^Department of Orthopedic Surgery, Thomas Jefferson University School of MedicinePhiladelphia, PA, USA; ^4^Uniformed Services University of the Health and Sciences, School of MedicineBethesda, MD, USA

**Keywords:** *Staphylococcus aureus*, *Staphylococcus epidermidis*, biofilm, phenol-soluble modulins, medical devices

## Abstract

Staphylococci are frequently implicated in human infections, and continue to pose a therapeutic dilemma due to their ability to form deeply seated microbial communities, known as biofilms, on the surfaces of implanted medical devices and host tissues. Biofilm development has been proposed to occur in three stages: (1) attachment, (2) proliferation/structuring, and (3) detachment/dispersal. Although research within the last several decades has implicated multiple molecules in the roles as effectors of staphylococcal biofilm proliferation/structuring and detachment/dispersal, to date, only phenol soluble modulins (PSMs) have been consistently demonstrated to serve in this role under both *in vitro* and *in vivo* settings. PSMs are regulated directly through a density-dependent manner by the accessory gene regulator (Agr) system. They disrupt the non-covalent forces holding the biofilm extracellular matrix together, which is necessary for the formation of channels, a process essential for the delivery of nutrients to deeper biofilm layers, and for dispersal/dissemination of clusters of biofilm to distal organs in acute infection. Given their relevance in both acute and chronic biofilm-associated infections, the Agr system and the *psm* genes hold promise as potential therapeutic targets.

## Introduction

First described in 1878, staphylococci are Gram-positive microorganisms that are implicated in human skin and soft tissue infections, blood stream infections including valvular and device-associated infective endocarditis, osteomyelitis, pneumonia, and infections involving other implanted medical devices (Lowy, 1998). Staphylococci are further classified based on coagulase designation, into coagulase-positive staphylococci, comprising mostly the important human pathogen *Staphylococcus aureus*, and the coagulase-negative staphylococci (CoNS) (Kloos and Schleifer, 1986). Of the CoNS, *Staphylococcus epidermidis* is most commonly isolated from human infections (Vuong and Otto, 2002). In the era of implantation of medical devices, many staphylococcal species have emerged as important pathogens, primarily due to their ability to form deeply seated microbial communities, referred to a biofilms, on the surfaces of native tissues and implanted medical devices (Costerton et al., 1999; Otto, 2008). Because these microbial communities are shielded from the effects of antimicrobial therapy and the host immune system, medical therapy involving infections of implanted medical devices can be particularly challenging (Hoiby et al., 2010). Often complete explantation of the implanted medical devices in conjunction with prolonged courses of antimicrobial therapy are necessary in curative approaches, incurring additional risks to patients and excess cost to the health care system.

Within the past decades, research efforts have led to important advances in the understanding of the molecular determinants of these microbial communities, implicating exopolysaccharides, proteins, and extracellular DNA (eDNA) in the formation of the extracellular biofilm matrix. Enzymes that degrade these molecules have been discussed as potential effector molecules of biofilm structuring and dispersal. However, much of these insights have been gleaned *in vitro*. Only recently have molecular tools enabled the optimization of *in vivo* models in the study of staphylococcal biofilm-associated infections (Joo and Otto, 2012). This review focuses on key *in vitro* and *in vivo* experiments that have led to current understanding of the determinants of staphylococcal biofilm structuring and dispersal.

## Stages of biofilm development

**Figure 1:**
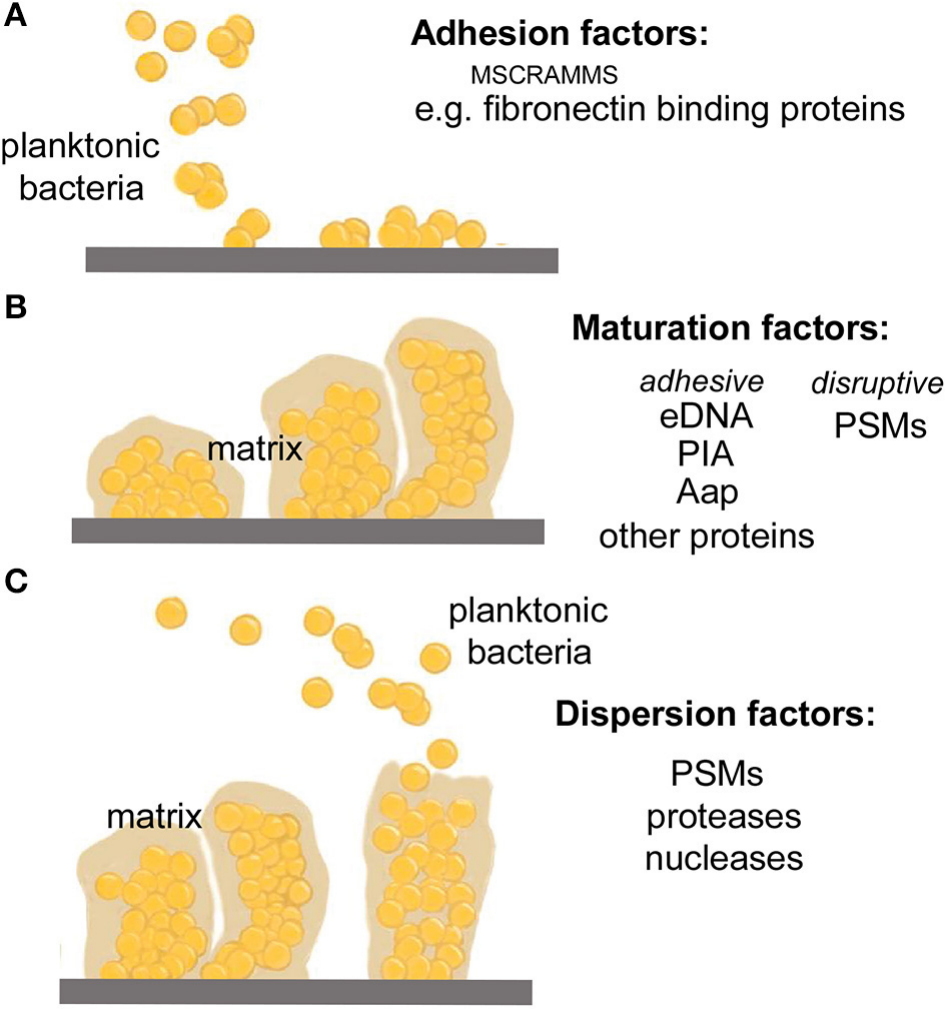
**Stages of biofilm development. (A)** During the attachment phase, planktonic bacteria adhere to a biotic surface, such as human tissue or a human matrix-covered indwelling device, by non-covalent interactions between human matrix proteins and dedicated bacterial surface binding proteins (mostly, MSCRAMMs). **(B)** After attachment is accomplished, biofilm cells multiply producing an extracellular biofilm matrix that is composed of a variety of macromolecules, including specific exopolysaccharides (in many staphylococci, PIA), eDNA, teichoic acids, and a series of proteins such as the fibril-forming accumulation-associated protein, Aap. Furthermore, the biofilm develops a structured form with channels and mushroom-like towers, which is dependent on the disruptive forces of the PSM structuring molecules discussed in this review. **(C)** In the last phase of biofilm development, clusters of bacteria or single bacteria may detach from the biofilm in a process also called dispersal or sloughing. This process is stimulated by mechanic forces (such as under flow), the PSM surfactants, and by enzymes that degrade biofilm matrix molecules such as nucleases and proteases. The relevance of the latter mechanism for infection is unclear.

Current literature models biofilm development in three stages: (1) attachment, (2) proliferation/formation of the matured biofilm, and (3) detachment/dispersal (O'Toole et al., 2000; Otto, 2013) (Figure 1). Based on work in *Pseudomonas aeruginosa*, these three stages have been further sub-categorized to include a total of five stages, but such further differentiation has not yet been made for staphylococcal biofilm development (Sauer et al., 2002). During attachment, staphylococcal surface-attached proteins, mostly so-called microbial components recognizing adhesive matrix molecules (MSCRAMMs) establish non-covalent interactions with host tissue or host protein that coat device surfaces (Patti et al., 1994; Otto, 2008). As discovered by *in vitro* research, several other surface molecules such as teichoic acids may also be important in the direct attachment to abiotic surfaces (Gross et al., 2001), which is, however, not believed to have an important role during the *in vivo* infection of indwelling medical devices. Following attachment, proliferation and maturation of the biofilm ensues, with the production of an extracellular matrix consisting of the staphylococcal biofilm exopolysaccharide polysaccharide intercellular adhesin (PIA) (Mack et al., 1996), teichoic acids, proteins and eDNA (Joo and Otto, 2012). During this stage, channels and mushroom-shaped structures form to facilitate nutrient delivery to deeper layers of the biofilm (O'Toole et al., 2000; Otto, 2008). The last stage of biofilm development is characterized by the detachment of biofilm clusters and the dissemination of these clusters to distal sites (O'Toole et al., 2000; Otto, 2008, 2013).

During the second and third stages of biofilm development, it is thought that disruption of intercellular adhesive forces is necessary for the formation of channels and mushroom-shaped structures, and also for biofilm detachment/dissemination (Otto, 2013). Previous work has implicated proteases (Boles and Horswill, 2008), nucleases (Mann et al., 2009; Sharma-Kuinkel et al., 2009; Kiedrowski et al., 2011; Beenken et al., 2012), and a family of staphylococcal proteins called phenol-soluble modulins (PSMs) (Wang et al., 2011; Periasamy et al., 2012b) in this role. However, of these proposed effector molecules, to date, only PSMs have been consistently demonstrated to facilitate staphylococcal biofilm maturation and dispersal through both *in vitro* and *in vivo* models (Otto, 2013).

## PSMs in biofilm structuring and dispersal

**Figure 2:**
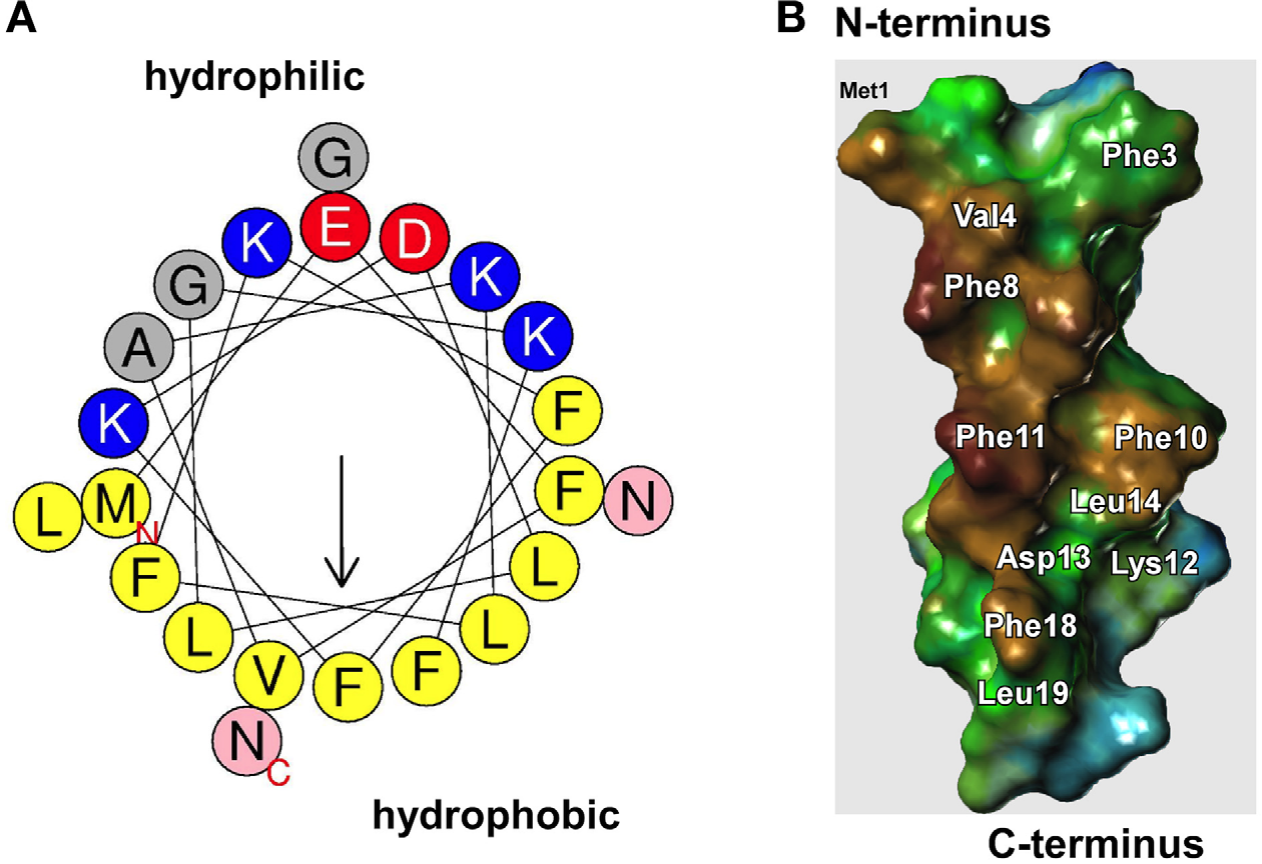
**Surfactant properties of PSMs. (A)** All PSMs form amphipathic α-helices. In the α-type PSMs, the helix stretches over virtually the whole peptide, while the longer β-type PSMs contain an α-helical part at their C-terminus. The graph shows an α-helical wheel presentation of PSMα3. Hydrophilic and hydrophobic amino acids occupy opposite sides of the helix, giving the helix strongly amphipathic character. **(B)** Model of PSMα3 structure (modeled after the known structure of δ-toxin that was determined by NMR studies). The hydrophobic side is shown. Replacement of amino acids on the hydrophobic side, mainly of large hydrophobic residues such as phenylalanine, leads to impaired biofilm structuring capacity.

First described in 1999 in *S. epidermidis* (Mehlin et al., 1999), PSMs are a family of small peptides (~21–44 amino acids long), with amphipathic, α-helical secondary structures and surfactant-like properties (Mehlin et al., 1999; Peschel and Otto, 2013; Cheung et al., 2014a) (Figure 2). The smaller, ~20 amino acid peptides are grouped into the α-class and the longer ~44 amino acid peptides into the β-class of PSMs. In particular the α-class peptides are cytotoxic to many cell types, and work by non-specific membrane damage, while the β-class peptides lack cytotoxicity (Wang et al., 2007). All PSMs are pro-inflammatory by activation of the formyl peptide receptor 2 (FPR2) on human immune cells (Wang et al., 2007; Kretschmer et al., 2010).

**Figure 3:**
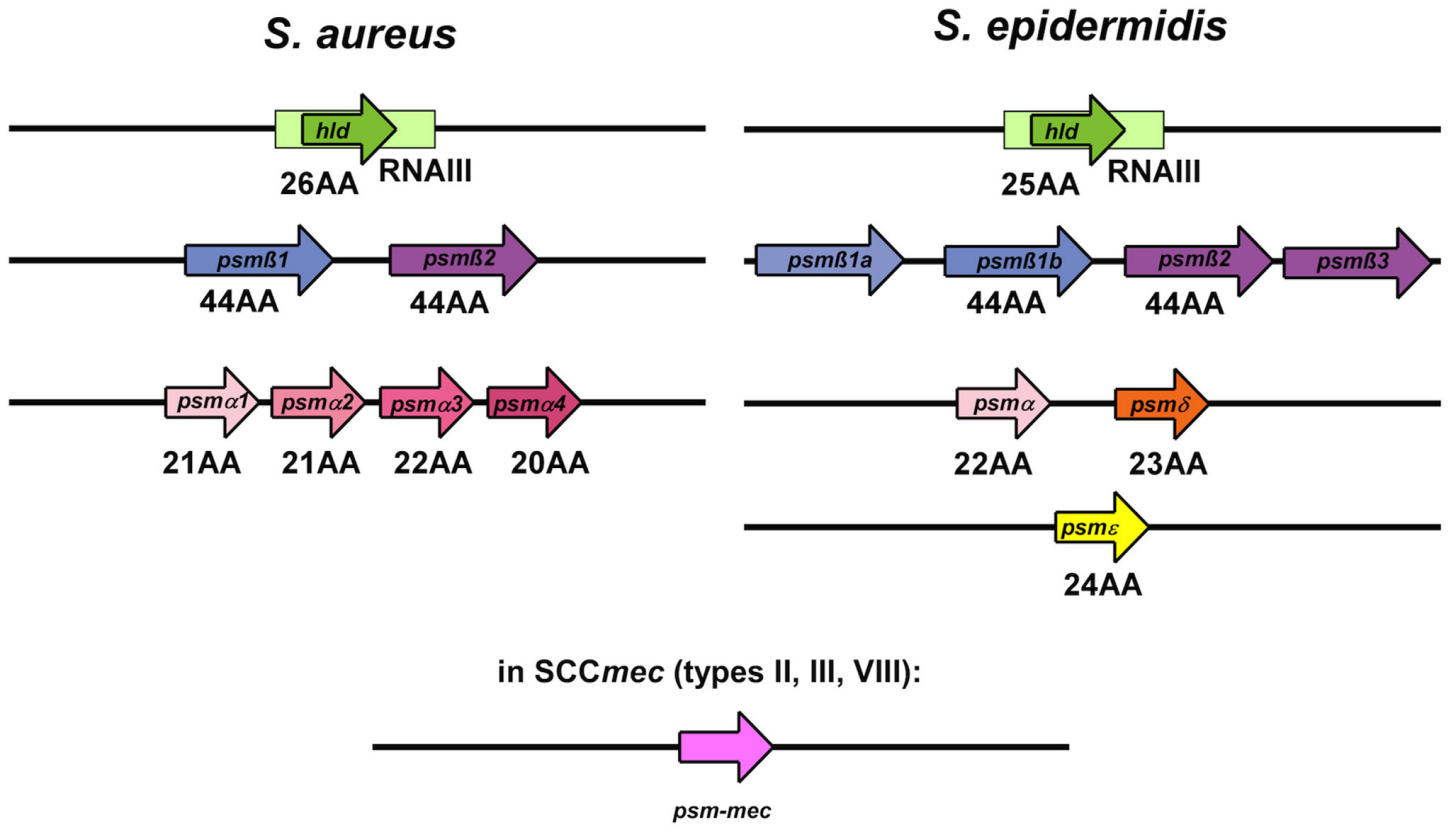
**PSM genes in *S. aureus* and *S. epidermidis***. PSMs are known to occur in a variety of staphylococci, but only in *S. aureus* and *S. epidermidis* were *psm* genes identified and gene products analyzed in a systematic manner. The graph shows the genetic arrangement of *psm* genes in *S. aureus* and *S. epidermidis*. The *hld*/RNAIII, *psm*β, and *psm*α/*psm*δ loci show strong similarity between *S. aureus* and *S. epidermidis*, suggesting that they are evolutionarily related. The *psm-mec* gene is encoded on SCC*mec* mobile genetic elements present in similar form in *S. aureus* and *S. epidermidis*.

*S. epidermidis* β-PSMs (Wang et al., 2011) and all of the *S. aureus* PSMs (Periasamy et al., 2012b) (Figure 3) have been shown to be key effector molecules in biofilm structuring and dissemination (Otto, 2013). Other *S. epidermidis* PSMs may have similar roles, but this is awaiting the rather difficult construction of *psm* gene deletion mutants in *S. epidermidis* and their investigation. The general mechanism by which PSMs contribute to biofilm structuring and dispersal is believed to be the disruption of non-covalent (electrostatic or hydrophobic) interactions between biofilm matrix macromolecules (Otto, 2013). While the physico-chemical properties of PSMs strongly favor that notion, direct evidence for such a mechanism is difficult to achieve. Some evidence is derived from an alanine screen of the PSMα3 peptide, whose capacity in biofilm detachment was most strongly impaired when large hydrophobic residues were exchanged for alanine (Cheung et al., 2014b) (Figure 2B). Notably, PSMs must be produced during biofilm growth for structuring and dispersal to take effect. External addition of PSMs to already formed biofilm does not disrupt biofilms (Wang et al., 2011), most likely because the physico-chemical mechanism by which PSMs work is not sufficient to disrupt the covalent bonds in macromolecular networks of, for example, exopolysaccharide that surrounds cells in a mature biofilm.

## *S. epidermidis* β-PSMs

*S. epidermidis* produces six PSM peptides, PSMα, PSMβ1, PSMβ2, PSMδ, PSMε, and δ-toxin (Wang et al., 2007). Next to δ-toxin, β-PSMs are the primary PSMs produced in *S. epidermidis* (Yao et al., 2005; Cheung et al., 2010), and seem to be key players in biofilm structuring and dispersal (Wang et al., 2011). They are encoded by the *psm*β operon, which also encodes a gene, *psm*β3, whose gene product does not appear to be produced or secreted (Yao et al., 2005; Cheung et al., 2010). Some strains have a duplication of the *psm*β1 gene, resulting in higher relative production of that PSMβ peptide. *In vitro*, the role of β-PSMs as effector molecules in *S. epidermidis* biofilm structuring and dispersal seems to be concentration-dependent (Wang et al., 2011). At medium concentrations, PSMβ peptides promoted biofilm formation, by providing the disruptive forces necessary for the formation of channels and thus formation of a mature biofilm. However, at higher concentrations, PSMβ caused biofilm detachment, thereby inhibiting overall biofilm extension. Of note, this mechanism was independent of the type of biofilm (protein- vs. exopolysaccharide-dependent) examined (Wang et al., 2011). This suggests that differential concentration allows the same effector molecule to play disparate roles in the earlier proliferative stage involving formation of the matured biofilm as well as the subsequent detachment/dispersal stage.

In a murine model of indwelling catheter-related infection, when compared to its isogenic *psm*β deletion mutant, the wild-type strain was noted to promote biofilm dissemination to the lymphatic system and the distal organs of the infected animals. Moreover, when compared to mice treated with control serum alone, those treated with anti-PSMβ antibodies had lower burden of dissemination of infection to their distal organs (Wang et al., 2011). These results support *in vitro* findings observed at higher concentrations of PSMβ, and recapitulate the role that PSMβ peptides play in *S. epidermidis* biofilm detachment and dissemination.

## *S. aureus* PSMs

*S. aureus* produces four PSMα peptides that are encoded in the *psm*α operon, two PSMβ peptides that are encoded in the *psm*β operon, and δ-toxin that is encoded by RNAIII (Wang et al., 2011; Peschel and Otto, 2013). When mutants in the *psm*α, *psm*β, and *hld* (the gene coding for δ-toxin) loci in clinically relevant community-associated methicillin-resistant *S. aureus* (CA-MRSA) strains (DeLeo et al., 2010) were examined in *in vitro* and *in vivo* models, all classes of *S. aureus* PSMs were implicated in biofilm structuring and detachment (Periasamy et al., 2012b). (The *hld* mutant was constructed by introducing an altered start codon, abolishing only translation of *hld*, not to interfere with the function of RNAIII.) *In vitro*, under both static and dynamic growth conditions, all isogenic *S. aureus psm* mutants produced thicker biofilms, demonstrated less channel formation, and had smoother surfaces than the wild-type (Periasamy et al., 2012b). It is remarkable that removal of any one class of PSMs resulted in a significant effect on the biofilm phenotype, indicating that presence of all PSMs is needed for efficient biofilm structuring and dispersal. Interestingly, the biofilm-enhancing effect was not additive, as in a complete *psm* deletion strain (*psm*α/*psm*β/*hld*), biofilm formation was not stronger than in the single *psm* deletion mutants, a result that yet remains unexplained. It may be due to the fact that in the absence of all PSMs, a beneficial effect on biofilm structuring and maturation, as seen with low concentrations of PSMβ peptides in *S. epidermidis* (Wang et al., 2011), is completely abolished. Furthermore, as in *S. aureus* PSMβ peptides are only produced at very low concentrations, the considerable impact on biofilm dispersal and structuring that was found with the *S. aureus psm*β deletion mutant is particularly remarkable (Periasamy et al., 2012b). Why biofilm dissemination/dispersal is thus most prominently seen within the β-subclass of PSMs remains unclear. It is plausible, but remains speculative, that since PSMβ is less cytotoxic than PSMα or the δ-toxin, the observed effects might be attributable to specialization of this subclass to the role of promotion of biofilm detachment/dissemination (Otto, 2013).

In murine models of *S. aureus* catheter infection, the isogenic *psm* triple deletion mutant (*psm*α/*psm*β/*hld*) demonstrated notably decreased infection dissemination to the surrounding tissues and the lymphatic system when compared to the wild-type strain (Periasamy et al., 2012b), confirming the role that PSMs play in the detachment/dispersal stage of *S. aureus* biofilm development. Here, in contrast to the *in vitro* results, the effect was additive, with the total *psm* deletion mutant showing a more pronounced phenotype than the single *psm* mutants. However, it needs to be stressed that in the case of the PSMα peptides, survival in organs may also be affected by their functions in evasion of innate host defense mechanisms.

## PSM-mec

PSM-mec is a PSM that—in contrast to all other characterized PSMs—is encoded on a mobile genetic element, namely staphylococcal cassette chromosomes (SCC) *mec* elements of types II, III, and VIII (Queck et al., 2009; Chatterjee et al., 2011). Its impact on biofilm formation *in vitro* is modest; *S. aureus psm-mec* mutants only show slightly decreased capacity to form biofilms and increased aggregation compared to the isogenic wild-type strain (Queck et al., 2009). These phenotypes are likely caused by a combination of the direct impact of PSM-mec on biofilm formation, which is negative (at concentrations in the physiological range of ~20–100 μg/ml), in accordance with that of other PSMs, and the negative impact that the *psm-mec* RNA has on the production of other *S. aureus* PSMs (Kaito et al., 2013; Cheung et al., 2014c).

## Do PSM fibrils have a role in *in vivo* biofilm formation?

It has been reported that some PSMs form amyloid-like fibrils *in vitro* and that *psm* mutants show less *in vitro* biofilm formation due to the lack of those fibrils (Schwartz et al., 2012). However, PSM fibrils were only observed in a specific growth medium. Notably, the theoretical impact that PSM fibrils have on the biofilm phenotype is essentially opposite to that facilitated by their biofilm-disruptive forces. *In vivo* results support the relevance of the latter mechanism during infection (Wang et al., 2011; Periasamy et al., 2012b), as described above.

## Regulation of PSMs by the Agr system

**Figure 4:**
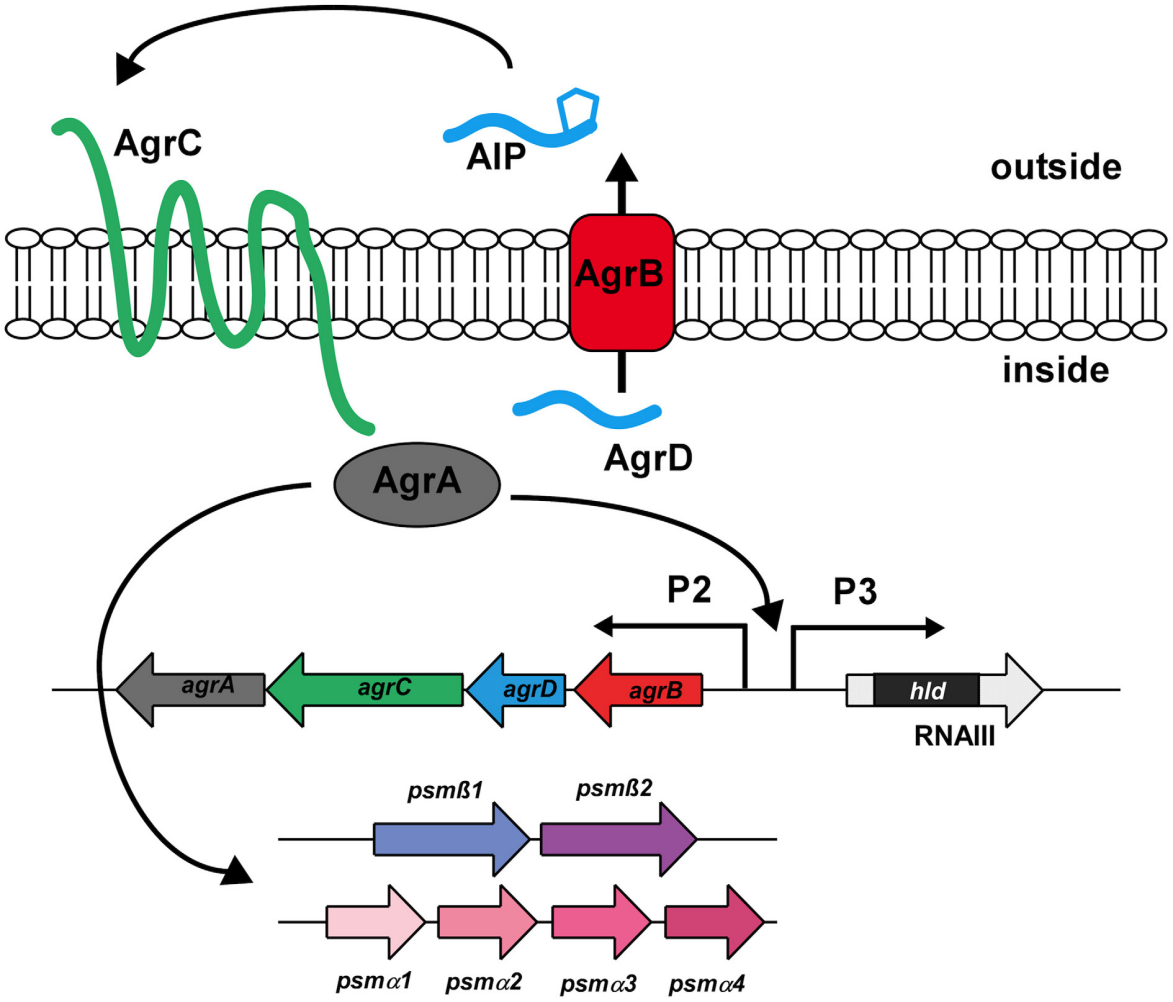
**The Agr quorum-sensing system**. The Agr system is an auto-regulatory system controlling gene expression in response to increasing cell density. It consists of the structural gene coding for the extracellular signal (AgrD), which is post-translationally modified and exported via AgrB. Upon reaching a certain threshold concentration, the AgrD AIP triggers auto-phosphorylation of the histidine kinase AgrC, which in turn leads to phosphorylation and activation of the DNA-binding response regulator AgrA. AgrA binding activates transcription from the AgrP2, AgrP3, *psm*α, and *psm*β promoters. Agr targets other than PSMs are regulated by RNAIII, the regulatory RNA surrounding the *hld* (δ-toxin) gene.

In staphylococci, the production of PSMs is controlled by the accessory gene regulator (Agr) system, a quorum-sensing mechanism that controls gene expression according to bacterial cell density (Novick et al., 1993; Vuong et al., 2004a; Wang et al., 2007). The *agr* locus contains the *agrA, C, D*, and *B genes* (RNAII transcript) and RNAIII which contains the *hld* gene that encodes the PSM δ-toxin (Novick et al., 1995) (Figure 4).

The Agr system regulates cell density-dependent gene expression using two proteins that comprise a classical two-component system, the sensor histidine kinase AgrC and the response regulator AgrA, and two proteins, AgrD and AgrB, which represent the structural and maturation proteins of the extracellular signal called auto-inducing peptide (AIP) (Novick and Geisinger, 2008). AIP binds to AgrC and activates (phosphorylates) the DNA-binding regulator AgrA, leading to the transcription of RNAIII and RNAII. This activation is dependent on the extracellular concentration of AIP, which signals cell density. As AIP thus promotes its own production, the circuit provides auto-feedback, leading to a rapid change of gene expression at a certain cell density. In contrast to all other targets of Agr, which include a series of positively regulated toxins and negatively regulated surface proteins that are controlled by RNAIII, expression of the *psm* operons is stimulated by direct binding of AgrA to their promoters (Queck et al., 2008). Control of PSMs by quorum-sensing thus likely preceded the link of other Agr targets to the system via the development of RNAIII around the gene encoding the PSM δ-toxin during evolution. This underlines the key role PSMs are believed to play both in the commensal and infectious lifestyles of staphylococci (Periasamy et al., 2012a).

Since the *psm* operons are under strict control by AgrA, *S. aureus* isogenic *agr* mutants have been shown to produce thicker biofilms (Vuong et al., 2000, 2003) and demonstrated less channel formation and smoother surfaces than the wild-type (Periasamy et al., 2012b). Furthermore, the phenotype of the total *S. aureus psm* deletion mutant (*psm*α/*psm*β/*hld*) was observed to be very similar to that of an *agr* mutant (Periasamy et al., 2012b). Moreover, expression of *agr* and *psm* were noted to be most prominent within the outer layers of the biofilm, the site of active biofilm expansion and dissemination. These findings indicate that PSMs are the key effector molecules of quorum-sensing dependent biofilm structuring and detachment in *S. aureus* (Periasamy et al., 2012b).

It is thought that up-regulation of the Agr system, leading to increased production of PSMs, favoring biofilm maturation and detachment, might have important roles in acute infection (Vuong et al., 2004a; Wang et al., 2007). Whereas, mutation of the Agr system or *psm* genes, favors the development of extensive and compact biofilms (Wang et al., 2011) that have lost the capacity to disseminate, a situation that may be of benefit in localized chronic biofilm-associated infections (Joo and Otto, 2012). In fact, *agr* mutants were frequently isolated from such infections (Vuong et al., 2004b; Traber et al., 2008).

Because in addition to the PSMs, the *agr* locus regulates a series of other cytotoxic toxins, such as leukocidins and α-toxin, it has been proposed that control of this locus might serve as a therapeutic target (Ji et al., 1997; Otto, 2004; Wright et al., 2005; Kong et al., 2006). However, the benefit of such intervention remains undefined with respect to biofilm-associated infections, as interference with *agr* inhibits dissemination of biofilm to distal targets (Wang et al., 2011) but also favors localized biofilm formation (Wang et al., 2011).

## Proteases and nucleases

Several proteins and eDNA have been implicated in *in vitro* staphylococcal biofilm formation; and thus, proteases and nucleases were found to contribute to biofilm structuring and dispersal *in vitro* (Boles and Horswill, 2008; Mann et al., 2009; Sharma-Kuinkel et al., 2009; Kiedrowski et al., 2011; Beenken et al., 2012). This is discussed in depth elsewhere in this review series. However, to date, *in vitro* findings have not been confirmed *in vivo*, or *in vitro* and *in vivo* findings have yielded conflicting results (Beenken et al., 2012). Furthermore, in contrast to PSM-mediated mechanisms, protease-dependent biofilm structuring is strain-dependent as proteins are premier determinants of biofilm formation only in a subset of strains (O'Neill et al., 2007). Therefore, the exact roles of degradative enzymes in staphylococcal biofilm structuring and dissemination/dispersal remain to be clearly delineated.

## Biofilm development: reflecting the “original role” of PSMs in the commensal lifestyle of staphylococci?

As, for example, the exceptionally direct mode of quorum-sensing control over PSM expression indicates, PSMs have a key and evolutionarily early role in staphylococcal pathogenesis (Queck et al., 2008; Periasamy et al., 2012a). The surfactant-based mechanism of biofilm structuring and detachment may be similar to the role that PSMs have during the commensal lifestyle of staphylococci on the human skin. In addition to structuring biofilm-like agglomerates in places like sebaceous glands, where staphylococci often reside, PSMs may also facilitate the acquisition of nutrients by emulsification and promote a means to spread over surfaces by surfactant-mediated “sliding” activity. On soft agar surfaces, PSMs have indeed been shown to promote such sliding activity (Tsompanidou et al., 2011, 2013).

## Conclusion

In summary, among the effector molecules that have been proposed as molecular determinants of staphylococcal biofilm dispersal and structuring, only PSMs have been demonstrated to be relevant in *S. aureus* and *S. epidermidis* biofilm-associated infection under both *in vitro* and *in vivo* settings. Under strict regulation by the global regulator Agr, PSMs are believed to enable the disruption of non-covalent forces in the biofilm matrix based on their amphipathic structure, to form channels that are necessary for the delivery of nutrients to deeper levels of the biofilm, and provide the disruptive forces necessary for the detachment of clumps of biofilm to distal sites.

### Conflict of interest statement

The authors declare that the research was conducted in the absence of any commercial or financial relationships that could be construed as a potential conflict of interest.
